# Empagliflozin and Dapagliflozin Improve Endothelial Function in Mexican Patients with Type 2 Diabetes Mellitus: A Double-Blind Clinical Trial

**DOI:** 10.3390/jcdd11060182

**Published:** 2024-06-15

**Authors:** Luis Ricardo Balleza Alejandri, Fernando Grover Páez, Erick González Campos, Carlos G. Ramos Becerra, Ernesto Germán Cardona Muñóz, Sara Pascoe González, María Guadalupe Ramos Zavala, Africa Samantha Reynoso Roa, Daniel Osmar Suárez Rico, Alberto Beltrán Ramírez, Jesús Jonathan García Galindo, David Cardona Müller, Claudia Yanette Galán Ruíz

**Affiliations:** 1 Department of Physiology, University Health Sciences Center, Universidad de Guadalajara, Guadalajara 44340, Mexico; luis.balleza3286@alumnos.udg.mx (L.R.B.A.); erick.gonzalez7206@alumnos.udg.mx (E.G.C.); carlos.rbecerra@academicos.udg.mx (C.G.R.B.); german.cardona@academicos.udg.mx (E.G.C.M.); sara.pascoe@academicos.udg.mx (S.P.G.); maria.ramos9950@alumnos.udg.mx (M.G.R.Z.); africa.reynoso0835@alumnos.udg.mx (A.S.R.R.); daniel.suarez@academicos.udg.mx (D.O.S.R.); alberto.beltran@academicos.udg.mx (A.B.R.); jonathan.garcia@academicos.udg.mx (J.J.G.G.); david.cardona@academicos.udg.mx (D.C.M.); claudia.galan3643@alumnos.udg.mx (C.Y.G.R.); 2 Arterial Stiffness Laboratory, Department of Physiology, Experimental and Clinical Therapeutics Institute, University Health Sciences Center, Universidad de Guadalajara, Guadalajara 44340, Mexico

**Keywords:** type 2 diabetes, empagliflozin, dapagliflozin, flow-mediated dilation

## Abstract

Aim: To assess the acute effect of empagliflozin versus dapagliflozin administration on flow-mediated vasodilation in patients with type 2 diabetes mellitus. Design: A double-blind clinical trial, at the Experimental and Clinical Therapeutics Institute, University Health Sciences Center, at the Universidad de Guadalajara, in inpatients with T2D according to the 2023 ADA criteria. Methods: Thirty patients (15 males and 15 females), aged between 35 and 65 years, were included in this study, according to the 2023 ADA criteria. The eligible patients were randomly assigned to three groups: empagliflozin 25 mg once daily, dapagliflozin 10 mg once daily, or placebo once daily. Anthropometric parameters were taken using validated techniques. FMD was measured using a high-resolution semiautomatic ultrasound UNEX-EF 38G (UNEX Co., Ltd., Nagoya, Japan). Arterial tension was determined with the OMRON electronic digital sphygmomanometer (HEM 907 XL, Kyoto, Japan). Results: The group of patients who received empagliflozin had a significantly lower baseline flow-mediated dilation (FMD) compared to the group receiving dapagliflozin (*p* = 0.017); at the end of this study, the empagliflozin group achieved a comparable FMD to the dapagliflozin group (*p* = 0.88). Conclusion: After the treatment period, the empagliflozin and dapagliflozin groups achieved similar FMD, suggesting a class effect.

## 1. Introduction

Type 2 Diabetes Mellitus (T2D) is a metabolic disorder of multiple etiologies and is considered one of the main global health challenges; according to data from the International Diabetes Federation (IDF) [1], it is estimated that, in 2021, there were 537 million people with diabetes, and this could increase to 643 million by 2030; in Mexico, according to the National Health and Nutrition Survey (ENSANUT 2018), approximately 10.3% of adults have already received a diagnosis of diabetes, and this may even be double, according to previous evidence, of the percentage of diabetics who do not know their condition [2].

Constant hyperglycemia in patients with diabetes is recognized as the main risk fac-tor for the development of macro- and microvascular complications in T2D, as it leads to damage to the vascular endothelium [3,4,5]. In addition to this, patients with type 2 diabetes mellitus T2D suffer from hypertension as the main comorbidity, being a major risk factor for the development of cardiovascular disease (CVD) (understood as angina, stroke, and heart failure) and microvascular complications, in addition to an increase in mortality [6,7,8].

Flow-mediated vasodilation (FMD) is the most widely used technique for the evaluation of endothelial function and is the most widely used non-invasive method, in which arterial diameter is measured before and after increasing wall stress induced by reactive hyperemia, resulting in the local release of nitric oxide. The lack of vasodilation suggests a decrease in the release of endogenous vasodilators and, consequently, endothelial dysfunction, which is accompanied by a decrease in shear stress. It has been found that FMD assessment can be used as a predictor of cardiovascular risk [9,10,11].

SGLT2 inhibitors (SGLT-2i) have been shown to decrease cardiovascular risk in patients with type 2 diabetes mellitus, decreasing arterial stiffness as well as endothelial dysfunction compared to a placebo and other agents [12,13,14,15]. Among the iSGLT2, empagliflozin has been approved by the FDA as a protective drug against CV risk. With this information available so far, it is unknown whether these acute hemodynamic benefits could correspond to a class effect or are specific to each of these drugs.

## 2. Materials and Methods

**Figure 1:**
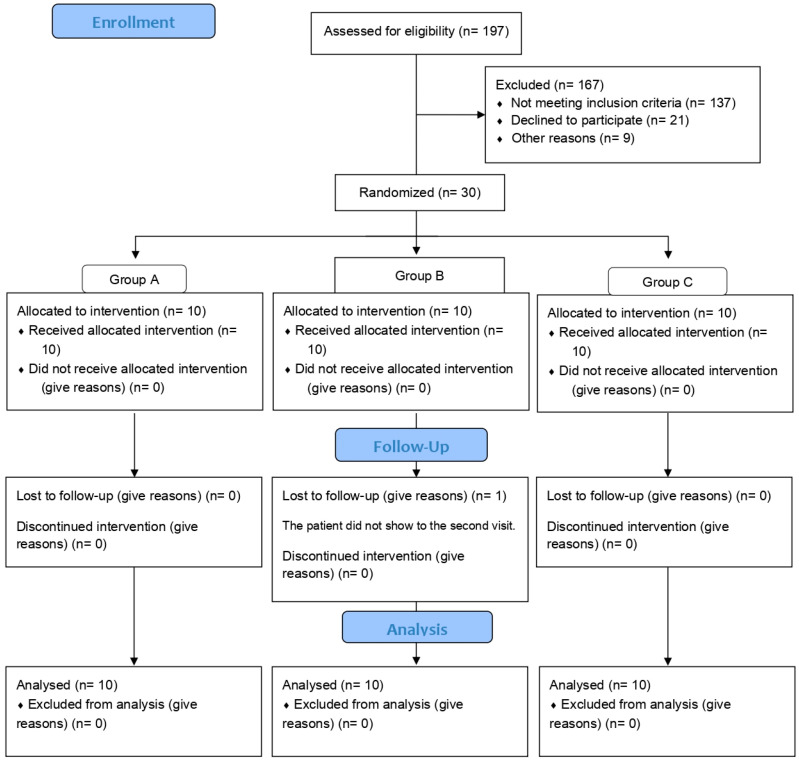
CONSORT flow diagram.

Design and subjects. A study flow diagram presenting the enrolment and randomization data is shown in Figure 1. A double-blind clinical trial was conducted in 30 patients (15 males and 15 females), included in this study according to the following criteria: (a) age between 35 and 65 years, (b) with a diagnosis of T2D according to the 2019 ADA criteria16, (c) HbA1c > 7 < 11%, (d) under stable hypoglycemic treatment for at least 6 months. Exclusion criteria were as follows: (a) Individuals with high blood pressure or on insulin treatment, (b) presence of thyroid disease, (c) presence of hypoglycemia in the previous 24 h, (d) presence of acute illness, (e) severe systemic disease, and (f) alcohol abuse. The trial took place at the Experimental and Clinical Therapeutics Institute (INTEC), Universidad de Guadalajara, México. Informed written consent was obtained from all subjects. All the subjects underwent a full medical history and physical examination, laboratory studies, and flow-mediated vasodilation. Blood samples were collected in the morning after an 8 h fasting period at baseline and after a 1-week intervention period. Eligible patients were randomized to receive empagliflozin (empagliflozin group) 25 mg once daily (OD) for 7 days or dapagliflozin (dapagliflozin group) 10 mg OD for 7 days or placebo (placebo group) OD for 7 days in a 1:1:1 ratio. Throughout this study, the subjects were instructed to continue their current treatment, diet, and physical activity, and a diary was given to the patients to report adverse effects.

Sample size determination. The sample size was calculated with a formula employing mean difference for clinical trials (Jeyaseelan and Rao 1989) [16], with a statistical confidence level of 95% and a statistical power of 80% and considering 1.21 (d) and 2.1 for standard deviation (SD) for FMD (Solini et al. 2017) [13]. A total of 30 patients were obtained, with an additional 20% for expected losses.

Statistical analysis. Values are expressed using the International System of Units. Quantitative variables are presented as mean and SD, while qualitative variables are presented as frequencies and percentages. The Shapiro–Wilk test was used to determine the normality of the data set. For non-parametric variables, we conducted a Kruskal–Wallis test; Wilcoxon’s signed-rank test was used to compare the results before and after the intervention, and a Dunn post hoc analysis was performed to compare the significance between groups. A *p*-value < 0.05 was considered statistically significant. All analyses were performed with SPSS v.25.0. (SPSS Inc., Chicago, IL, USA).

Clinical determinations. Anthropometric measurements, including height, body weight, and body mass index (BMI), were measured and conducted in duplicate using validated measurement techniques with the individuals wearing light clothes. Height was measured using a height rod with patients standing without shoes, and the measurements were rounded to the nearest centimeter. Body weight was measured with a bioimpedance digital scale (TBF-215^®^ Body Composition Analyzer, Tanita Corporation, Tokyo, Japan). BMI was calculated using the formula BMI = kg/m^2^. Venous blood samples were obtained and centrifuged at 2500 rpm (Beckman Coulter, Allegra X-22R, Brea, CA, USA). Serum and plasma were separated and stored at −80 °C for analysis.

Blood pressure. An OMRON-calibrated electronic digital sphygmomanometer model HEM 907 XL was used. The patients were under sedation on a chair, resting their backs on it, with a minimum rest of 5 min and their right and left arms uncovered. They were asked to place their left arm on a table at the height of their heart and their right arm on their knee, while keeping both feet resting on the floor, without crossing them. A bracelet was placed on the circumference of the left arm and fitted 3 cm above the antecubital fold. Three measurements were taken and recorded with an interval of 1 min, from which the average taken as the TA value was obtained. Values are reported in mmHg.

Flow-mediated dilation. The measurement of the FMD was carried out in the morning, with the individuals in a fasting state. Following the protocol of the guidelines for FMD11, the individual was placed in a supine position, in a semi-dark room with a constant temperature of 22 °C. Using a calibrated electronic digital sphygmomanometer from the brand OMRON, model HEM 7320, the bracelet was placed on the circumference of the left arm and fitted 3 cm above the antecubital fold. Using the high-resolution semiautomatic ultrasound UNEX-EF 38G (UNEX Co., Ltd., Nagoya, Japan) with a linear transducer at 10 MHz, longitudinal images of the right brachial artery were obtained continuously for 30 s. The bracelet was inflated (50 mmHg over systolic pressure) for 5 min, after which the bracelet was slowly deflated for 2 min. The diameter of the brachial artery was determined using the software UUSM-PCALSS-00104 v 8.20.32 included in the device at 60 and 71 s. The adventitious and intima-media segments were determined automatically and followed automatically. FMD was estimated as the percentage of the relationship between the maximum change in diameter during reactive hyperemia over basal diameter.

## 3. Results

The present study included 30 patients, 15 men, and 15 women, with an average age of 49.9 ± 6.47 with a previous diagnosis of T2D. The three groups were comparable in terms of age, sex, BMI, HbA1c percentage, triglycerides, SBP, SBP, minimum and maximum diameter, average flow, and bIMT, but not in glucose and FMD, as summarized in Table 1.

As summarized in Table 2, according to the Kruskal–Wallis test, we found significant differences in endothelial function between the study groups (*p* 0.031, 0.010). Table 2 summarizes the demographic and hemodynamic characteristics of the patients after the 7-day intervention.

After obtaining significant FMD results from the Kruskal–Wallis test, we found significant differences when performing a post hoc Dunn analysis between the dapagliflozin and empagliflozin groups in baseline measurements (*p* 0.049), but not in final measurements (*p* 1.000). In the final basal comparison between the groups, we found significant differences between the placebo and empagliflozin (*p* < 0.001) groups and between the empagliflozin and dapagliflozin (*p* 0.015) groups, but none between the placebo and dapagliflozin (*p* 0.134) groups (Table 3).

On the other hand, when comparing empagliflozin vs. dapagliflozin, the effect on FMD appears to be a class effect; however, comparing the deltas, empagliflozin seems to be superior to dapagliflozin.

According to this, more studies are required to support these findings.

### Adherence and Safety

No serious adverse events were reported in either treatment arm. The adherence to dapagliflozin and empagliflozin, estimated by the number of pills returned at each visit, was 99% and 100%, respectively. Safety was evaluated with the personal diaries given to the patients. There were two reported cases of headache, and only symptomatic treatment was necessary in both cases; no other adverse effects were reported.

## 4. Discussion

Constant hyperglycemia in patients with diabetes is recognized as the main risk factor for the development of macro- and microvascular complications in T2D, leading to endothelial damage, with a consequent decrease in nitric oxide production, which plays an important role in vascular dysfunction, leading to an increased risk of mortality in these patients, highlighting the importance of treating such damage in these patients [5,17,18].

Several experimental studies have reported that SGLT2i shows vasodilatory effects both dependent and independent of the endothelium. Initially, it was considered that empagliflozin had a vasodilatory effect which dapagliflozin did not have [19]; however, in subsequent studies (Li et al., 2018) [20], it was found that dapagliflozin induced vasodilation through different mechanisms, without involving endothelium through the activation of PKG and voltage-dependent potassium channels (Kv) [20]. In addition to decreasing proinflammatory cytokines, increasing the expression of vascular adhesion molecules (Gaspari et al., 2018) [15], and chronically improving endothelial function (Lee et al., 2018) [21], they also directly inhibit the cardiac output of the Na/H pump, reducing cytosolic Na, and mediate the attenuation of TNF-α (Uthman, 2017) [22].

It has been reported that the use of SGLT2i leads to a decrease in the development of cardiovascular complications in patients with T2D, reducing arterial stiffness, reducing endothelial dysfunction, and increasing shear stress and blood viscosity; it has also been reported that SGLT2i can lead to a reduction in the monocyte/high-density lipoprotein (HDL) ratio (MHR), decreasing the inflammation and oxidative stress, thus leading to both renoprotective and cardioprotective effects [14,23]. Moreover, it has been shown that after 2 days of treatment with dapagliflozin, patients had a significant increase in FMD of 1.2% (from 2.8 to 4.0%), while in our study, we found an improvement of 0.91% (from 6.51 to 7.42%) in the group of patients receiving dapagliflozin, whereas in the group of patients receiving empagliflozin, the improvement was 2.6% (from 4.62 to 7.22%).

According to these results, it seems that the benefit obtained by patients receiving empagliflozin is higher than that by patients receiving dapagliflozin; however, we must consider that there was a significant difference in baseline values between both groups, as demonstrated in Dunn’s post hoc analysis, and this difference was lost after pharmacological intervention. Based on these results, we can theorize that the greater the endothelial dysfunction, the greater the potential short-term effects of SGLT2i on reducing this endothelial dysfunction. These findings are consistent with the report by Tahara et al. (2017) [24], showing experimentally that the use of SGLT2i could be useful not only for hyperglycemia but also for diabetes-related diseases and complications.

The main limitation of our study may be the limited sample size of patients, although we identified notable results. We believe it is crucial to examine the impact of SGLT-2i on changes in endothelial function during different treatment periods to confirm our findings.

## 5. Conclusions

After 7 days of treatment, both the empagliflozin group and dapagliflozin group achieved similar FMD effects, suggesting that these results may be considered as a class effect on endothelial function.

## Figures and Tables

**Table 1:** Baseline demographic and hemodynamic characteristics.

	Placebo	Dapagliflozin	Empagliflozin	*p* *
Age	49 ± 4.02	52.30 ± 6.99	48.40 ± 7.72	0.267
Sex, female/male	7/3	5/5	6/4	
BMI, kg/m^2^	30.8 ± 3.26	28.67 ± 3.44	30.23 ± 3.83	0.527
Glucose, mg/dL	176.90 ± 59.80	175.97 ± 40.65	227.22 ± 65.13	0.018
HbA1c, %	8.91 ± 0.94	8.1 ± 0.81	8.9 ± 0.83	0.101
TG, mg/dL	140.57 ± 34.10	145.80 ± 41.68	131.89 ± 33.71	0.759
SBP, mmHg	120.90 ± 8.5	126.70 ± 11.79	127.60 ± 21.35	0.587
DPB, mmHg	79.10 ± 6.26	87.10 ± 20.25	82.30 ± 10.38	0.490
FMD, %	5.10 ± 1.97	6.51 ± 1.52	4.62 ± 1.32	0.031
Minimum diameter, mm	3.66 ± 0.80	3.77 ± 0.45	4.62 ± 1.24	0.100
Maximum diameter, mm	3.83 ± 0.87	3.72 ± 0.36	4.08 ± 0.87	0.686
Average Flow, L	4.91 ± 2.16	4.69 ± 1.96	4.50 ± 2.48	0.867
bIMT, mm	0.27 ± 0.12	0.29 ± 0.07	0.26 ± 0.06	0.119

BMI: Body mass index. HbA1c: Glycosylated hemoglobin A1C. TG: Triglycerides. SBP: Systolic blood pressure. DBP: Diastolic blood pressure. FMD: Flow-mediated dilation. bIMT: Brachial intimate-media thickness. * Kruskal–Wallis test was performed.

**Table 2:** Demographic and hemodynamic characteristics after 7-day treatment.

	Placebo	Dapagliflozin	Empagliflozin	*p* *
Age	49 ± 4.02	52.30 ± 6.99	48.40 ± 7.72	0.267
Sex, female/male	7/3	5/5	6/4	-
BMI, %	30.6 ± 3.48	28.67 ± 3.44	29.36 ± 4.01	0.527
Glucose, mg/dL	177.39 ± 56.79	136.10 ± 40.64	170.70 ± 39.17	0.058
TG, mg/dL	234.56 ± 34.10	145.80 ± 41.68	109.97 ± 11.83	0.042
SBP, mmHg	116.4 ± 7.67	108.97 ± 18.32	116.18 ± 10.04	0.741
DBP, mmHg	77.20 ± 5.34	73.38 ± 7.31	73.30 ± 8.17	0.436
FMD, %	5.02 ± 1.95	7.42 ± 1.55	7.22 ± 1.63	0.010
Minimum diameter, mm	3.84 ± 0.80	4.02 ± 0.46	4.08 ± 0.86	0.083
Maximum diameter, mm	4.12 ± 0.96	4.00 ± 0.38	4.30 ± 0.90	0.765
Average Flow, L	4.91 ± 1.88	5.48 ± 1.35	5.65 ± 3.21	0.764
bIMT, mm	0.30 ± 0.11	0.28 ± 0.04	0.28 ± 0.04	0.561

BMI. Body mass index. HbA1c: Glycosylated hemoglobin A1C. TG: Triglycerides. SBP: Systolic blood pressure. DBP: Diastolic blood pressure. FMD: Flow-mediated dilation. bIMT: Brachial intimate-media thickness. * Kruskal–Wallis test was performed.

**Table 3:** FMD group comparison basal (A), final (B), and change (C) after 7-day treatment with dapagliflozin, empagliflozin, or placebo.

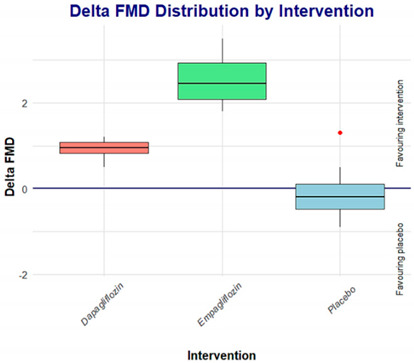									
	A			B			C		
	Test Statistic	Std Test Statistic	*p* *	Test Statistic	Std Test Statistic	*p* *	Z	*p*.unadj	*p adj* *
Placebo-Dapagliflozin	−8.4	−2.13	0.098	−10.6	−2.695	0.021	2.007049	0.045	0.045
Placebo-Empagliflozin	1.05	0.267	1.000	−10.1	−2.567	0.031	4.814376	<0.001	<0.001
Empagliflozin-Dapagliflozin	9.45	0.016	0.049	0.5	0.127	1.000	−2.807327	0.005	0.007

* The Dunn post hoc analysis; significance values were adjusted by Bonferroni correction. FMD: flow-mediated dilation.

## Data Availability

Data are contained within the article.

## References

[1] International Diabetes Federation Diabetes Atlas (2017). IDF Diabetes Atlas. http://www.diabetesatlas.org/.

[2] Hernández-Ávila M., Gutiérrez J.P., Reynoso-Noverón N. (2013). Diabetes mellitus in Mexico. Status of the epidemic. Salud Publica Mex..

[3] El-Daly M., Venu V.K.P., Saifeddine M., Mihara K., Kang S., Fedak P.W., Alston L.A., Hirota S.A., Ding H., Triggle C.R. (2018). Hyperglycaemic impairment of PAR2-mediated vasodilation: Prevention by inhibition of aortic endothelial sodium-glucose-co-Transporter-2 and minimizing oxidative stress. Vasc. Pharmacol..

[4] Fowler M.J. (2008). Microvascular and Macrovascular Complications of Diabetes. Clin. Diabetes.

[5] Papatheodorou K., Banach M., Edmonds M., Papanas N., Papazoglou D. (2015). Complications of Diabetes. J. Diabetes Res..

[6] Park S., Kang H.-J., Jeon J.-H., Kim M.-J., Lee I.-K. (2019). Recent advances in the pathogenesis of microvascular complications in diabetes. Arch. Pharmacal Res..

[7] ElSayed N.A., Aleppo G., Aroda V.R., Bannuru R.R., Brown F.M., Bruemmer D., Collins B.S., Das S.R., Hilliard M.E., Isaacs D. (2022). 10. Cardiovascular Disease and Risk Management: Standards of Care in Diabetes—2023. Diabetes Care.

[8] ElSayed N.A., Aleppo G., Aroda V.R., Bannuru R.R., Brown F.M., Bruemmer D., Collins B.S., Cusi K., Hilliard M.E., Isaacs D. (2022). 4. Comprehensive Medical Evaluation and Assessment of Comorbidities: Standards of Care in Diabetes-2023. Diabetes Care.

[9] Anderson E.A., Mark A.L. (1989). Flow-mediated and reflex changes in large peripheral artery tone in humans. Circulation.

[10] Gori T., von Henning U., Muxel S., Schaefer S., Fasola F., Vosseler M., Schnorbus B., Binder H., Parker J.D., Münzel T. (2016). Both flow-mediated dilation and constriction are associated with changes in blood flow and shear stress: Two complementary perspectives on endothelial function. Clin. Hemorheol. Microcirc..

[11] Corretti M.C., Anderson T.J., Benjamin E.J., Celermajer D., Charbonneau F., Creager M.A., Deanfield J., Drexler H., Gerhard-Herman M., Herrington D. (2002). Guidelines for the Ultrasound Assessment of Endothelial-Dependent Flow-Mediated Flow-Mediated Vasodilation of the Brachial Artery. The American College of Cardiology. J. Am. Coll. Cardiol..

[12] Cefalu W.T., Leiter L.A., de Bruin T.W., Gause-Nilsson I., Sugg J., Parikh S.J. (2015). Dapagliflozin’s effects on glycemia and cardiovascular risk factors in high-risk patients with type 2 diabetes: A 24-week, multicenter, randomized, double-blind, placebo-controlled study with a 28-week extension. Diabetes Care.

[13] Solini A., Giannini L., Seghieri M., Vitolo E., Taddei S., Ghiadoni L., Bruno R.M. (2017). Dapagliflozin acutely improves endothelial dysfunction, reduces aortic stiffness and renal resistive index in type 2 diabetic patients: A pilot study. Cardiovasc. Diabetol..

[14] Irace C., Casciaro F., Scavelli F.B., Oliverio R., Cutruzzolà A., Cortese C., Gnasso A. (2018). Empagliflozin influences blood viscosity and wall shear stress in subjects with type 2 diabetes mellitus compared with incretin-based therapy. Cardiovasc. Diabetol..

[15] Gaspari T., Spizzo I., Liu H., Hu Y., Simpson R.W., Widdop R.E., Dear A.E. (2018). Dapagliflozin attenuates human vascular endothelial cell activation and induces vasorelaxation: A potential mechanism for inhibition of atherogenesis. Diabetes Vasc. Dis. Res..

[16] Jeyaseelan L., Rao P.S. (1989). Methods of determining sample sizes in clinical trials. Indian Pediatr..

[17] American Diabetes Association (2019). 2. Classification and Diagnosis of Diabetes: Standards of Medical Care in Diabetes—2019. Diabetes Care.

[18] Chen L., Magliano D.J., Zimmet P.Z. (2012). The worldwide epidemiology of type 2 diabetes mellitus—Present and future perspectives. Nat. Rev. Endocrinol..

[19] Uthman L., Baartscheer A., Bleijlevens B., Schumacher C.A., Fiolet J.W.T., Koeman A., Jancev M., Hollmann M.W., Weber N.C., Coronel R. (2018). Class effects of SGLT2 inhibitors in mouse cardiomyocytes and hearts: Inhibition of Na+/H+ exchanger, lowering of cytosolic Na+ and vasodilation. Diabetologia.

[20] Li H., Shin S.E., Seo M.S., An J.R., Choi I.-W., Jung W.-K., Firth A.L., Lee D.-S., Yim M.-J., Choi G. (2018). The anti-diabetic drug dapagliflozin induces vasodilation via activation of PKG and Kv channels. Life Sci..

[21] Lee D.M., Battson M.L., Jarrell D.K., Hou S., Ecton K.E., Weir T.L., Gentile C.L. (2018). SGLT2 inhibition via dapagliflozin improves generalized vascular dysfunction and alters the gut microbiota in type 2 diabetic mice. Cardiovasc. Diabetol..

[22] Uthman L., Baartscheer A., Schumacher C.A., Fiolet J.W.T., Kuschma M.C., Hollmann M.W., Coronel R., Weber N.C., Zuurbier C.J. (2018). Direct Cardiac Actions of Sodium Glucose Cotransporter 2 Inhibitors Target Pathogenic Mechanisms Underlying Heart Failure in Diabetic Patients. Front. Physiol..

[23] Tekin B.G., Pektaş E. (2024). Investigation of MHR-nephropathy relationship and the effect of SGLT2is on MHR in patients with type 2 diabetes. Ir. J. Med. Sci..

[24] Tahara A. (2021). SGLT2 inhibitor ipragliflozin exerts antihyperglycemic effects via the blood glucose-dependent increase in urinary glucose excretion in type 2 diabetic mice. Eur. J. Pharmacol..

