# Correction: Balleza Alejandri et al. Empagliflozin and Dapagliflozin Improve Endothelial Function in Mexican Patients with Type 2 Diabetes Mellitus: A Double-Blind Clinical Trial. *J. Cardiovasc. Dev. Dis.* 2024, *11*, 182

**DOI:** 10.3390/jcdd11100314

**Published:** 2024-10-09

**Authors:** Luis Ricardo Balleza Alejandri, Fernando Grover Páez, Erick González Campos, Carlos G. Ramos Becerra, Ernesto Germán Cardona Muñóz, Sara Pascoe González, María Guadalupe Ramos Zavala, Africa Samantha Reynoso Roa, Daniel Osmar Suárez Rico, Alberto Beltrán Ramírez, Jesús Jonathan García Galindo, David Cardona Müller, Claudia Yanette Galán Ruíz

**Affiliations:** 1 Department of Physiology, University Health Sciences Center, Universidad de Guadalajara, Guadalajara 44340, Mexico; luis.balleza3286@alumnos.udg.mx (L.R.B.A.); erick.gonzalez7206@alumnos.udg.mx (E.G.C.); carlos.rbecerra@academicos.udg.mx (C.G.R.B.); german.cardona@academicos.udg.mx (E.G.C.M.); sara.pascoe@academicos.udg.mx (S.P.G.); maria.ramos9950@alumnos.udg.mx (M.G.R.Z.); africa.reynoso0835@alumnos.udg.mx (A.S.R.R.); daniel.suarez@academicos.udg.mx (D.O.S.R.); alberto.beltran@academicos.udg.mx (A.B.R.); jonathan.garcia@academicos.udg.mx (J.J.G.G.); david.cardona@academicos.udg.mx (D.C.M.); claudia.galan3643@alumnos.udg.mx (C.Y.G.R.); 2 Arterial Stiffness Laboratory, Department of Physiology, Experimental and Clinical Therapeutics Institute, University Health Sciences Center, Universidad de Guadalajara, Guadalajara 44340, Mexico

In the original publication [1], there was a mistake in Table 3 as published. The specific conclusion of the study was that both empagliflozin and dapagliflozin were superior in their effect on endothelial function, as measured by FMD, compared to placebo. However, as indicated in Table 3, no statistically significant differences were observed between the delta of dapagliflozin vs. placebo, which seems to suggest that the results reported in the table do not fully support this conclusion. Considering this, the authors decided to conduct a reanalysis of the results and found that there is indeed a statistically significant difference when comparing the deltas of dapagliflozin vs. placebo, the corrected Table 3 appears below.

On the other hand, when comparing empagliflozin vs. dapagliflozin, the effect on FMD appears to be a class effect; however, comparing the deltas, empagliflozin seems to be superior to dapagliflozin.

According to this, more studies are required to support these findings.

The authors state that the scientific conclusions are unaffected. This correction was approved by the Academic Editor. The original publication has also been updated.

## Figures and Tables

**Table 3:** FMD group comparison basal (A), final (B), and change (C) after 7-day treatment with dapagliflozin, empagliflozin, or placebo.

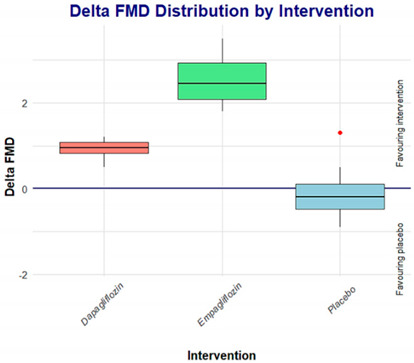									
	A			B			C		
	Test Statistic	Std Test Statistic	*p* *	Test Statistic	Std Test Statistic	*p* *	Z	*p*.unadj	*p adj* *
Placebo-Dapagliflozin	−8.4	−2.13	0.098	−10.6	−2.695	0.021	2.007049	0.045	0.045
Placebo-Empagliflozin	1.05	0.267	1.000	−10.1	−2.567	0.031	4.814376	<0.001	<0.001
Empagliflozin-Dapagliflozin	9.45	0.016	0.049	0.5	0.127	1.000	−2.807327	0.005	0.007

* The Dunn post hoc analysis; significance values were adjusted by Bonferroni correction. FMD: flow-mediated dilation.
